# The PDZ-Binding Motif of HPV16-E6 Oncoprotein Modulates the Keratinization and Stemness Transcriptional Profile* In Vivo*

**DOI:** 10.1155/2017/7868645

**Published:** 2017-10-10

**Authors:** Enoc Mariano Cortés-Malagón, Carmen Palacios-Reyes, Sandra Romero-Cordoba, Daniel Mendoza-Villanueva, Jaime Escobar-Herrera, Odette Verdejo-Torres, Rubén Gerardo Contreras, Gloria Fernádez-Tilapa, Mario Adan Moreno-Eutimio, José Moreno, Alfredo Hidalgo-Miranda, Patricio Gariglio, José Bonilla-Delgado

**Affiliations:** ^1^Genetics and Cancer Research Unit, Hospital Juárez de México (HJM), 07760 Mexico City, Mexico; ^2^Oncogenomic Department, Instituto Nacional de Medicina Genómica (INMEGEN), 14610 Mexico City, Mexico; ^3^Laboratory of Cell and Developmental Signaling, Center for Cancer Research, National Cancer Institute, Frederick, MD 21702, USA; ^4^Department of Cellular Biology, Centro de Investigación y de Estudios Avanzados (Cinvestav), 07360 Mexico City, Mexico; ^5^Department of Physiology, Biophysics, and Neurosciences, Centro de Investigación y de Estudios Avanzados (Cinvestav), 07360 Mexico City, Mexico; ^6^Laboratory of Clinic Research, Unidad Académica de Ciencias Químico-Biológicas, Universidad Autónoma de Guerrero (UAGro), 39070 Chilpancingo de los Bravo, GRO, Mexico; ^7^Department of Genetics and Molecular Biology, Centro de Investigación y de Estudios Avanzados (Cinvestav), 07360 Mexico City, Mexico

## Abstract

**Objective:**

The aim of this work was to compare the early gene expression profiles in the skin of HPV16-E6 transgenic mice regulated by the E6 PDZ-binding motif.

**Materials and Methods:**

The global transcriptional profiles in dorsal skin biopsies from K14E6 and K14E6Δ146-151 transgenic mice were compared using microarrays. Relevant genes obtained from the most differentially expressed processes were further examined by RT-qPCR, in situ RT-PCR, Western blot, or immunofluorescence.

**Results:**

The transcriptomic landscape of K14E6 versus K14E6Δ146-151 shows that the most affected expression profiles were those related to keratinocyte differentiation, stem cell maintenance, and keratinization. Additionally, downregulation of epidermal stemness markers such as K15 and CD34, as well as the upregulation of cytokeratin 6b, appeared to be dependent on the E6 PDZ-binding motif. Finally, wound healing, a physiological process linked to stemness, is impaired in the K14E6 mice compared to K14E6Δ146-151.

**Conclusion:**

The E6 PDZ-binding motif appears to affect stemness and keratinization during early stages of skin carcinogenesis. As E6 plays a significant role in HPV-induced skin carcinogenesis, the K14E6 versus K14E6Δ146-151 transcriptional profile provides a source of valuable data to uncover novel E6 functions in the skin.

## 1. Introduction

Human papillomaviruses (HPVs) are small double-stranded DNA viruses that are the main factors for cervical intraepithelial neoplasia, warts, and, less commonly, nonmelanoma skin cancers (NMSCs) [1]. To produce precancerous lesions, the HPV infects the basal stratum of injured stratified epithelia at a variety of anatomical sites. Once the HPV genome has entered the cell, early HPV oncogenes synergize with environmental cofactors to induce cellular immortalization and tumour growth [2]. Most of the HPV oncogenic potential is mediated by E6 and E7 oncogenes, whose protein products block cellular differentiation and induce cell proliferation by mechanisms that are, as yet, not fully understood [3].

Contrary to cervical cancer, much less is known about NMSCs caused by HPV infections. The most frequent NMSCs lesions caused by HPV infections are epidermoid carcinomas, which in the majority of the cases are associated with HPV beta genus, particularly the HPV5 and 8 genotypes [4]. Although HPV-alpha genus rarely infects the nonmucosal skin, HPV16 infections are also found in epidermoid carcinomas in the perianal skin [5], and experimental data on K14E6 transgenic mice suggest that the sole expression of the E6-HPV16 oncoprotein in the basal stratum suffices to generate grade I–III epidermoid carcinomas in the dorsal skin of 1-year-old mice [6]. Interestingly, the HPV16-E6 oncogenic potential in the skin can be virtually ablated* in vivo *if a truncated version of the E6 oncoprotein, lacking the PDZ-binding motif (K14E6Δ146-151 mice), is expressed in the basal stratum [7].

Considering that the E6 PDZ-binding motif appears to play a significant role in HPV-induced skin carcinogenesis, we compared the transcriptional landscapes of skin biopsies from K14E6 versus K14E6Δ146-151. Our results suggest that keratinization and stemness are two of the most differentially affected processes dysregulated by the E6 PDZ-binding motif. The knowledge of novel genes affected by E6 HPV16 oncoprotein will lead us to a better understanding of HPV-induced skin carcinogenesis.

## 2. Materials and Methods

### 2.1. Sample Isolation of K14E6 and K14E6Δ146-151 Strains

The construction of transgenic mice expressing the full version of the HPV16-E6 oncogene (K14E6), or its truncated version lacking the E6 PDZ-binding motif (K14E6Δ146-151), has been described previously [6, 7]. The transgenic strains and nontransgenic strain (NTG-FvB/N) were housed and treated according to the American Association of Laboratory Animal Care (AALAC) regulations, and the Research Unit approved the research protocol for Laboratory Animal Care Committee (UPEAL-CINVESTAV-IPN, Mexico; NOM-062-ZOO-1999). Skin biopsies from 1.5-month-old transgenic and NTG mice were resected, fixed (paraformaldehyde 4%, 1x PBS), and paraffin-embedded for histological procedures, or they were immediately frozen in liquid nitrogen for protein or RNA isolation (TRIzol reagent, Ambion®). For histological procedures, 5 *μ*m thick dorsal skin transversal sections were mounted on charged microscope slides (Fisher Scientific®) for immunofluorescence, or in situ RT-PCR techniques. For wound healing experiments, excisional 2 mm punches were made with a metal ear puncher on ears (28 days after wounding, to evaluate wound closure), or in shaved dorsal skin (72, 96, and 120 hours after wounding, to evaluate reepithelization), and visualised by H&E staining. For wound closure assays, the diameter of the hole openings was measured at 28 days after punching under a dissecting microscope using a micro ruler.

### 2.2. Microarray Procedure

K14E6 and K14E6Δ146-151 mice strains were sacrificed by cervical dislocation, and an excisional biopsy from shaved skin (0.5 cm^2^) was taken immediately and stored in RNA later solution (Ambion) at 4°C overnight. Standard procedures performed the RNA extraction, and the integrity was evaluated using an Agilent 2100 Bioanalyzer (Agilent Technologies®). Only samples with an Integrity Number greater than 8.0 were considered for microarray procedures. The RNA from three different mice was pooled, and two independents pools were used by each strain (each microarray experiment was performed as biological duplicates). The expression of 14000 genes was analysed using a Mouse Genome 430A 2.0 microarray (Affymetrix®). Background correction (RMA) and quantile normalisation were performed with the Affy package included in Bioconductor of R software. To define significant expression between the biological conditions, a moderate *t*-test was computed with Limma package implemented in Bioconductor. The biological significance of the altered genes (log⁡FC > 1.5 and adj *p* value < 0.05) was assayed by a pathway analysis with InnateDb database, David database, and ClusterProfiles module of R using gene ontology (Biological processes), Kegg, and Reactome annotations terms. The microarray data were deposited in the NCBI GEO database (GEOID: GSE99868) and validated by analysing the expression of 7 different genes by RT-qPCR as described below.

### 2.3. RT-qPCR

To validate microarray data, we analysed SFN, FOXN1, Krt15, LHX2, CD34, and SOX9 gene expression by using mouse-predesigned oligos (Assay IDs: Mm.PT.58.42262048.g, Mm.PT.58.13135783, Mm.PT.58.5528981, Mm.PT.58.6480133, Mm.PT.58.8626728, and Mm.PT.58.42739087, resp., IDT®) and K6b-designed oligos (5′-CATCAAATACACCACCAGCG-3′ (forward) and 5′-AAGCAGCCAAAAAGAGAAGC-3′ (reverse)). The quantitative real-time PCR (RT-qPCR) was carried out using a LightCycler 2.0 apparatus (Roche®) and a DNA Master SYBR Green I kit (Roche). The templates were amplified in 45 cycles of a 3-step PCR process, which included 30 seconds of a denaturation step at 95°C, a 30-second primer-dependent annealing phase (60°C), and a 30-second template-dependent elongation at 72°C. The amplification of each template was conducted in duplicate in one PCR run. The differential expression of each mRNA was calculated as a ratio normalised to glyceraldehyde-3-phosphate dehydrogenase (GAPDH) gene expression. The data were analysed using the equation that was previously described by Livak and Schmittgen (amount of target = 2^−ΔΔCt^ [8]).

### 2.4. In Situ RT-PCR

Dried dewaxed skin sections were incubated in a protein lysis buffer (0.1 M Tris–HCl pH: 8.0, 50 mM EDTA pH 8.0) containing 0.5 g/ml Proteinase K for 30 min at room temperature. After thorough washing with DEPC-treated water, 50 *μ*l of the PCR master mix solution containing digoxigenin-11-(20-deoxy-uridine-50)-triphosphate (DIG-11-dUTP; Roche) was added. Negative controls were made without primers or Taq. In situ, PCR was performed using the system provided by Perkin Elmer®. The slides were preheated to 70°C on the assembly tool included in the in situ Perkin Elmer equipment. 50 l PCR master mix was added to each sample, and the reaction was sealed using AmpliCover discs and clips (Perkin Elmer). After assembly, slides were placed at 70°C in the GeneAmp in situ PCR system 1000 (Perkin Elmer) until running was started. PCR amplification was performed running 18 cycles using the K6b designed oligos. The same primers were used for end-point RT-PCR and RT-qPCR. After cycling had been completed, the temperature was kept at 4°C until disassembly. Clips were removed, and AmpliCover discs were very carefully lifted from the slides without moving them sideways, and slides were washed for 5 min in 1x PBS followed by 5 min in 100% EtOH before they were air-dried. Slides were soaked in PBS containing 5% bovine serum albumin (Sigma®) for 30 min to block nonspecific binding activity (stringent wash). Immunohistochemical signal detection was carried out using mouse anti-digoxigenin monoclonal antibody Fab fragments conjugated to alkaline phosphatase (1 : 200 dilution, 30 min, room temperature) (Roche), and signals were visualised by nitro blue tetrazolium chloride (NBT) and bromochloroindoxyl phosphate (BCIP) (Zymed®).

### 2.5. Immunofluorescence

The skin sections were deparaffinized and rehydrated as described previously [6]. Briefly, tissue sections were rinsed in 1x PBS, and the epitope retrieval was performed in a pressure cooker for 12 min for a subsequently tissue-blockade with 1x PBS supplemented with 0.3% Triton X-100 and 1% bovine serum albumin. K15, CD34, *β*-catenin, and E-cadherin (Santa Cruz Biotechnology®) primary antibodies were incubated each at 4°C overnight in a humid chamber and then incubated with a fluorescein isothiocyanate (FITC-labeled), or tetramethylrhodamine (TRITC-labeled) secondary antibody (Zymed) for 1 h at room temperature; they were rinsed above and mounted in Vectashield (Vector®). The preparations were examined by confocal microscopy using an SP2 (Leica Microsystems®). Captured images were imported into the ImageJ® software program (version 1.37v, NIH, Bethesda, MD) to produce maximum projections, and Adobe Photoshop (Adobe Systems®) was used to equalise the brightness and contrast in all of the images. The positive signals in the immunofluorescence images were quantified from 3 different animals per experiment (amplification: 20–40x; 3 sections per mouse) using the Image-Pro Plus 7.0 software program (Media Cybernetics®), and Student's statistical *t*-test was conducted using the SPSS 20.0 software package (IBM®).

### 2.6. Western Blot

Skin biopsies were subjected to total protein extraction, and 20 *μ*g was used for SDS-PAGE and transferred to an immobilon-P membrane (Millipore®). The membrane was blocked with 5% nonfat dry milk in 1 TBS (20 mmol/L Tris–HCl, pH 7.5, 150 mmol/L NaCl, and 0.5% Tween-20) for 1 h, blotted with an anti-CD34 (1 : 2000) primary antibody overnight, and then incubated with a horseradish peroxidase- (HRP-) linked secondary antibody (GE Healthcare®) for 1 h. The membranes were developed using the Millipore Immobilon Western Chemiluminescent HRP Substrate according to the manufacturer's instructions. Chemiluminescence was detected using a FujiFilm LAS-3000 imaging system.

## 3. Results

### 3.1. The E6 PDZ-Binding Motif Suppresses Keratinocyte Differentiation, Stem Cell Maintenance, and Keratinization Transcriptional Profiles

**Figure 1:**
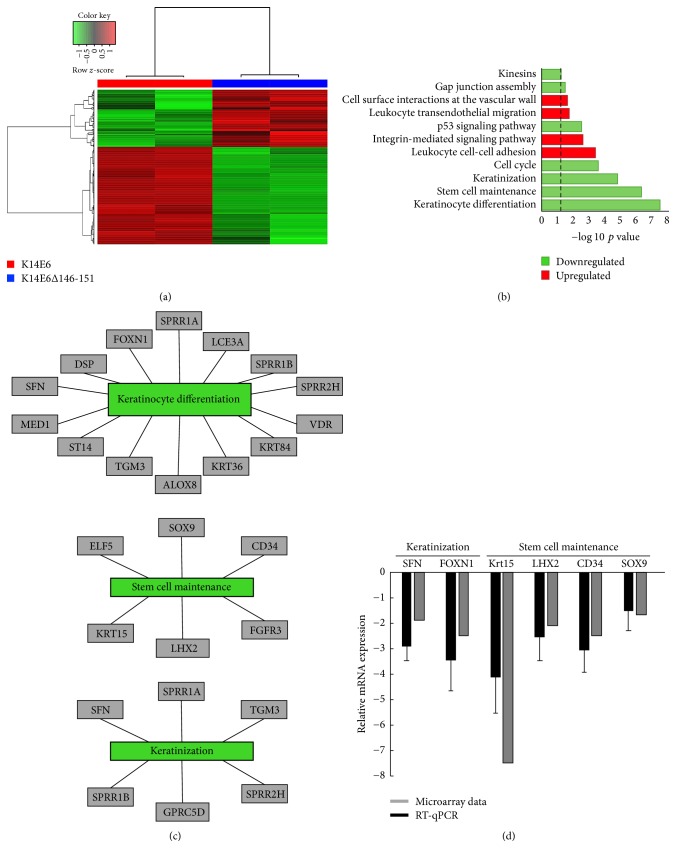
Global transcriptional analysis of K14E6 versus K14E6Δ146-151 mice. (a) Hierarchical clustering of genes differentially expressed in an RNA-microarray analysis performed on K14E6 versus K14E6Δ146-151 comparison. A *z*-score adjusts heatmap. (b) Pathway enrichment plot of differentially expressed genes. The plot shows the significantly (−log⁡10  *p* value) upregulated (red bars) and downregulated (green bars) pathways of differentially expressed genes. (c) Networks of three of the most negatively enriched cellular processes (keratinocyte differentiation, stem cell maintenance, and keratinization) based on statistical significance (−log⁡10  *p* value). (d) Microarray validation by RT-qPCR. Grey bars represent microarray expression value versus black bars which represent RT-qPCR validation; each RT-qPCR validation was performed as *n* = 3 biological replicates.

The K14E6 transgenic mice gradually develop epidermoid carcinomas (grade I–III) when they reach 1 year of age [6]. Contrary to the above, K14E6Δ146-151 mice do not develop epidermoid carcinomas through their life cycle [7]. To gain insights about these phenotypical discrepancies, we performed an expression microarray assay in skin biopsies resected from younger adult mice (1.5-month-old). From a total of 14,000 genes, 324 genes were differentially expressed in the skin of K14E6 mice as compared to K14E6Δ146-151. From those, 204 were downregulated, and 120 were upregulated (see Supplementary Figure  1 in Supplementary Material available online at https://doi.org/10.1155/2017/7868645). Heatmap analysis (Figure 1(a)) clearly shows that differentially expressed genes can be clustered accordingly to their pattern expression in each mouse strain.

We next search for cellular processes derived from the microarray data. By performing a Pathway Enrichment Analysis, Figure 1(b) shows that the most significant changes occur in genes related to keratinocyte differentiation, stem cell maintenance, and keratinization processes, all of them, significantly downregulated. Additionally, Figure 1(c) shows the most negatively enriched genes for each of the three most differentially affected processes. Finally, we validate microarray data by the RT-qPCR analysis of 6 biologically relevant genes, which shows the same expression tendency (Figure 1(d)).

### 3.2. The E6 PDZ-Binding Motif Enhances the Expression of Cytokeratin 6b

Cytokeratins are structural elements implicated in skin's mechanical resistance [9]. Their pattern/abundance are well-known and are routinely used as cellular markers to evaluate the normal keratinization process in several stratified epithelia [10]. Typically, basal K5/K14 cytokeratins are gradually replaced by K1/K10 when keratinocytes reach the spinous stratum [9, 11]. However, under a wound stimulus, basal keratinocytes express K6b/K16 instead of K1/K10 in response to cytokines and immune mediators released during wound repair [12].

**Figure 2:**
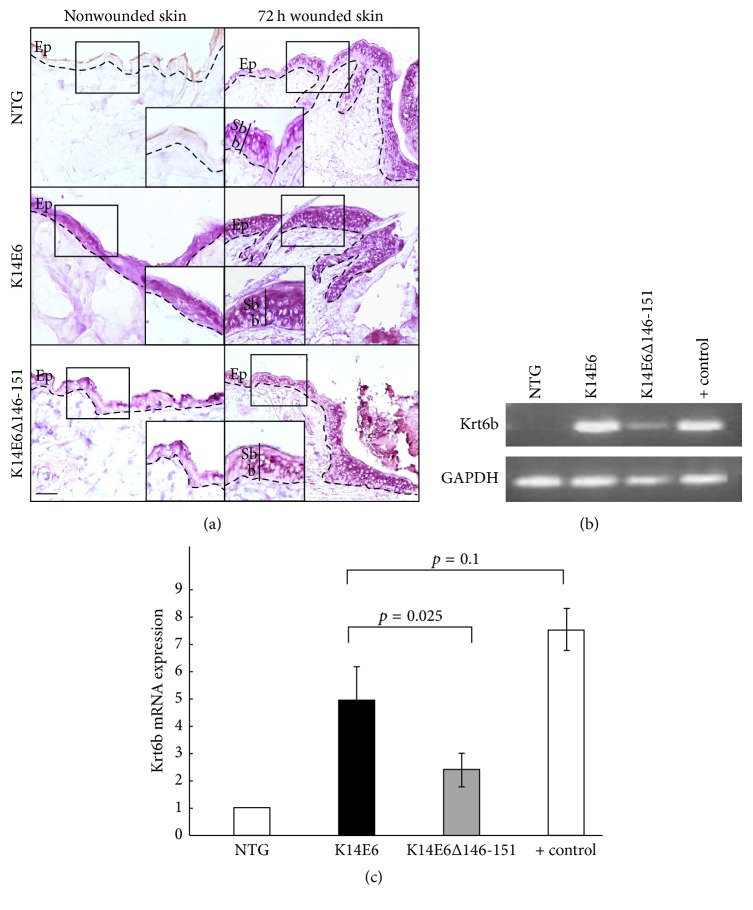
Expression and cellular localisation of keratin 6b mRNA. (a) In situ RT-PCR showing the cellular localisation of mRNA in nonwounded, and 72 h wounded dorsal skin evaluated in 1.5-month-old NTG, K14E6, and K14E6Δ146-151 transgenic mice. Magnification: 20x. Dashed line: basal membrane. Ep: epidermis, Sb: suprabasal stratum; and b: basal stratum. (b) End-point RT-PCR showing the amplification products of K6b and GAPDH genes. (c) The RT-qPCR assay is showing the relative expression of Krt6 using the amount of target = 2^−ΔΔCt^ equation; Krt6 RT-qPCR was performed as *n* = 3 biological replicates. Positive control for (b) and (c) refers to 72 h wounded skin samples. Magnification: 20x.

As Krt6 gene shows an upregulated pattern in our microarray data (see Supplementary Figure  1), we were interested in comparing mRNA abundance and cellular localisation in K14E6 and K14E6Δ146-151 skin samples. As Figure 2(a) shows, nonwounded NTG mice lack Krt6b mRNA expression; however, a wound stimulus can induce its expression in the suprabasal stratum as reported [12]. Contrary to NTG mice, Krt6 expression in K14E6 mice seems not to depend on wound stimulus, and wounded K14E6 mice still retain a ubiquitous Krt6b expression in the entire epidermis (second column, middle panel, Figure 2(a)). Concerning K14E6Δ146-151 mice, the histological expression of Krt6 resembles more the NTG phenotype than K14E6. Moreover, Krt6b relative expression levels in K14E6Δ146-151 mice are lower than K14E6 mice but slightly higher as compared to NTG mice (see Figures 2(b) and 2(c)). Therefore, the E6-HPV16 oncoprotein potentiates Krt6 mRNA expression, and it probably depends on its PDZ-binding motif.

### 3.3. The E6 PDZ-Binding Motif Delocalizes E-Cadherin Protein in the Skin

**Figure 3:**
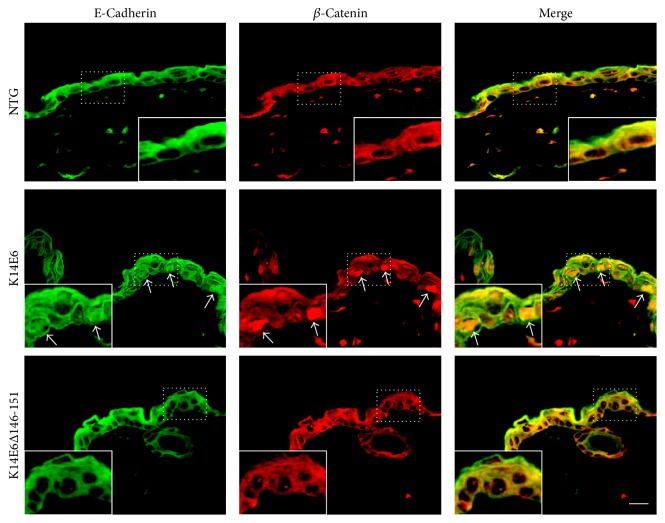
E-Cadherin and *β*-catenin cellular localisation in the skin. Immunofluorescence assay of nonwounded skin showing the expression and cellular localisation of E-cadherin (FITC-signal) or *β*-catenin (TRITC-signal) proteins in 1.5-month-old NTG, K14E6, and K14E6Δ146-151 transgenic mice. Insets highlight the nuclear signal indicated by arrows. Magnification: 40x.

The Krt6 gene is frequently induced in wound edges in which cell-cell contacts, such as adherent junctions, are lost [13], and E6 PDZ-binding motif binds and degrades scaffold proteins that participate in adherent junctions formation [14, 15]. As E-cadherin and *β*-catenin cellular localisation is a useful criterion to evaluate adherent junctions, we next wondered whether the E6 PDZ-binding motif relates to an impaired architecture of adherent junctions. As Figure 3 shows, E-cadherin immunofluorescent localisation displays a diffuse pattern with few cells exhibiting a nuclear signal in 1.5-month-old K14E6 mice as compared to NTG or K14E6146-151 mice (in which both show signal at the cell membrane) (Figure 3, first column). Additionally, *β*-catenin, a protein that normally colocalizes with E-cadherin at the cell membrane, also shows a diffusive localisation pattern, with few cells showing nuclear signal (see Figure 3, second column). Therefore, we suggest that the E6 PDZ-binding motif could impair the localisation of proteins related to adherent junctions, such as E-cadherin or B-catenin, in 1.5-month-old E6 transgenic mice.

### 3.4. The E6 PDZ-Binding Motif Suppresses K15 and CD34 Epidermal Stem Cell Markers and Impairs Reepithelization and Wound Closure Processes

**Figure 4:**
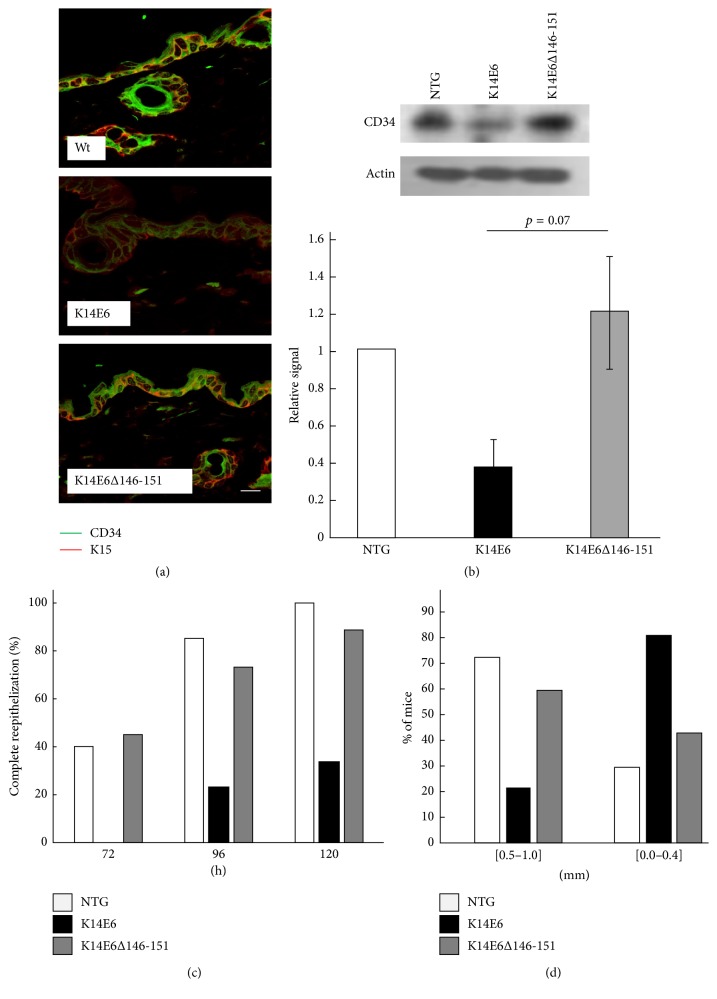
CD34/Krt15 epidermal stemness markers and reepithelization/wound closure assays. (a) Immunofluorescence assay of nonwounded skin showing the expression and colocalization of CD34 (FITC-signal) and K5 (TRITC-signal) stemness markers. (b) Western blot of CD34 evaluated in total protein extracts of nonwounded skin samples. The graph shows the result of *n* = 4 independent biological replicates. (c) Epithelization assay performed in 72, 96, and 120 h wounded dorsal skin indicating the percentage of complete epithelization for each mice strain. (d) Wound closure measure 28 days after wounding evaluated on ears. The hole diameter was measured with a micro ruler using a stereoscopic microscope. Magnification of (a): 40x.

As Figure 1(c) indicates, epidermal stemness markers such as K15 and CD34 were also differentially downregulated in K14E6 versus K14E6Δ146-151 mice microarray data. Epidermal stemness markers include integrin B1, p63, CD34, and Krt15 and are used, altogether with holoclone expansion assays, to evaluate the stem cells population in the skin [16]. We, therefore, validated the K15 and CD34 protein expression in E6 transgenic mice by immunofluorescence.  Figure 4(a) indicates that CD34 and K15 epidermal stem cell markers diminish their expression only in the K14E6 mice, as well as CD34 signal evaluated by immunoblot (Figure 4(b)). We also wondered whether this reduction in epidermal stemness markers could impair physiological processes linked to stemness such as wound healing. In general, wound healing process initiates by covering the wound area with migrating keratinocytes (reepithelization), followed by their proliferation and wound contraction (wound closure) [17]. Although both, reepithelization and wound closure, present some variability in each mice strain, there is a clear association between the presence of the E6 PDZ-binding domain and the skin capacity to reepithelize or close 2 mm wound holes.  Figure 4(c) indicates that complete reepithelization can be seen from the 72 h postwounded dorsal skin for NTG or K14E6Δ146-151 mice, and near 100% of the analysed samples reach complete epithelization at 120 h after wounding. However, although K14E6 manages to be completely epithelized, it clearly shows a delayed reepithelization at 120 h after wounding. Finally, we measured wound closure in 28-day postwounded ears; Figure 4(d) and Supplementary Figure  2 show that a wound reduction of 0.5–1.0 mm can be seen in 70% of NTG mice, 58% of K14E6Δ146-151, and 21% of K14E6 mice, demonstrating that wound closure can also be affected by the E6 PDZ-binding motif.

## 4. Discussion

The K14E6 mice develop spontaneous epidermoid carcinomas (grades I–III) in the skin of 1-year-old mice [6], but the precise mechanism by which the HPV16-E6 oncoprotein generates NMSC lesions is unknown. Contrary to K14E7, the K14E6 mice are more prone to develop spontaneously malign tumours in the skin of 1-year-old mice, or in 6-month-old mice after 5 months of DMBA + TPA treatment. By using chemical carcinogens, Song and coworkers demonstrated that around 75% of K14E6 mice developed grade III epidermoid carcinomas as compared to only 11% of K14E7 mice [18]. Interestingly, the K14E6Δ146-151 mice, which express a truncated form of HPV16-E6 lacking the PDZ-binding motif, not only fail to develop spontaneous skin cancer at 1-year aged animals [7] but also exhibit a similar frequency to developed skin cancer as NTG-FvB/N mice after DMBA + TPA treatment [19].

Although the E6 PDZ-binding motif has experimental evidence to support its relevance in skin carcinogenesis, other HPV-targeted epithelia, such as the cervix, seem not to be affected by the E6 oncogene (in comparison to E7 oncogene) [20]. As an approach to explaining this discrepancy, we reported the transcriptomic analysis of skin versus cervix tissue of K14E6 mice. We demonstrated that the HPV16-E6 oncogene is more prone to dysregulate the global transcriptional profile in skin samples than cervical tissue and that differentially dysregulated genes are involved in processes such as the cell cycle, apoptosis, the immune response, angiogenesis, cell junctions, and keratinocyte differentiation [21].

It is known that HPV-related skin cancers are not as frequent as cervical cancer, but there is a subset of cutaneous beta-genus HPVs (i.e., HPV5 and HPV8) that naturally infects the skin and provokes epidermoid carcinomas [4], and in vivo models such as K14HPV8 transgenic mice suggest that the sole expression of the HPV8 early region on basal stratum suffices to develop NMSC without any treatment with physical or chemical carcinogens [22]. To characterise the role of individual E6/E7 oncogenes in skin carcinogenesis, Marcuzzy and coworkers separated HPV8-E6/E7 oncogenes in transgenic mice. Nearly all K14-HPV8-E6 mice developed multifocal tumours characterised by papillomatosis, hyperkeratosis, and varying degrees of epidermal dysplasia, and only K14-HPV8-E6 benign tumours progress to NMSC [23]. In addition to K14-HPV8-E6 mice, the K14-HPV16-E6 mice, in particular the E6 PDZ-binding motif, do develop NMSC [6], and although HPV-alpha genus rarely infects the nonmucosal skin, its presence in HPV-associated skin lesions is frequent in the perianal skin [5]. Thus, at least for murine epidermis, experimental evidence suggests that HPV-E6 is the major oncogene necessary and sufficient to induce spontaneous tumour development up to the level of NMSC epidermoid carcinomas.

The K6b induction is associated with migratory keratinocytes at wound edges, but we observe that K14E6 mice exhibited an impaired reepithelization and wound closure phenotype (see Figures 4(c) and 4(d)). There is little data about abnormal Krt6 expression and its consequences on wound healing; however, Wong and coworkers demonstrated that, contrary to what is expected, cultured keratinocytes from null Krt6 mice exhibited enhanced reepithelization in in vitro assays [24], and wound closure in embryonic mouse skin is significantly delayed only in Krt17 null embryos, but not Krt6 null embryos [13]. Therefore, it seems that alterations in cytokeratins genes impair the compromise between cell migration and cell migration resilience during wound healing.

Finally, the deficient wound healing phenotype exhibited by the K14E6 mice (Figure 4) could also be explained by the sustained overexpression of the c-Myc. We reported previously that the K14E6 mice activate the canonical Wnt pathway in the skin, as well as Wnt targets genes such as c-Myc, CCND1, and BIRC5 in nonwounded skin biopsies [15]. The consequences of c-Myc overactivation in the epidermal stem cells population were already conducted by other groups [25–27]. They demonstrated that the constitutive expression of c-Myc in the skin commits the epidermal stem cells to differentiating into terminal amplifying cells. They evaluated the epidermal stem cell reservoir by assessing epidermal stem cell markers, as well as the holoclone expansion, and they found that c-Myc overexpression diminishes stem cell population and, as expected, wound healing processes were also impaired in K14-c-Myc mice [27]. Therefore, Wnt pathway activation in K14E6 mice could also impair wound healing through its target gene c-Myc.

## 5. Conclusion

Keratinocyte differentiation, stemness, and keratinization are candidate processes that could be early dysregulated by the E6 PDZ-binding motif during skin carcinogenesis. However, their real implications in HPV-induced skin carcinogens need to be further addressed.

## Supplementary Material

Supplementary Figure 1 (Caption): A complete list of differentially expressed genes from K14E6 vs. K14E6d146-151 mice transcriptional profiles.
Supplementary Figure 1 (Figure text): The list shows differentially up- or down-regulated genes resulted from global transcriptional microarray experiments.
Supplementary Figure 2 (Caption): Histogram of wound closure of ears.
Supplementary Figure 2 (Figure text): The histogram shows the distribution frequencies of wound closure every 0.1 mm in the ears of each mice strain. NTG: 24 samples, K14E6: 28 samples, and K14E6d146-151: 12 samples.
