# Supplementary material for: The PDZ-Binding Motif of HPV16-E6 Oncoprotein Modulates the Keratinization and Stemness Transcriptional Profile In Vivo
Source: Biomed Res Int. 2017 Oct 10;2017:7868645. doi: 10.1155/2017/7868645 (PMC5654334; doi:10.1155/2017/7868645)

Supplementary Figure 1

Downregulated genes							
Gene	EntrzId	logFC	adj.P.Val	B	AveExpr	t	P.Value
Krtap16-3	71369	-9.6597836	0.00695334	0.58699227	7.43797898	-6.6167814	0.00032859
Krtap15	26560	-9.5031783	0.00332978	2.82096919	7.66821437	-9.2803702	3.99E-05
Gm10228	100040214	-9.4944663	0.01572577	-1.297946	8.23195738	-4.8882067	0.00188869
Krtap6-5	68484	-9.2381591	0.0186795	-1.6685198	8.10552969	-4.5879719	0.00266308
Krtap19-2	170651	-9.1003726	0.00248703	3.58133219	7.0646023	-10.403417	1.91E-05
Krt34	16672	-9.0262597	0.00734219	0.45652192	8.3804265	-6.4844087	0.00037104
Gm10229	100040201	-9.0079875	0.0151188	-1.2189557	9.01085185	-4.9536506	0.00175538
Krt33a	71888	-8.8306955	0.00664031	0.70298856	8.21571062	-6.7363001	0.00029492
Krtap6-1	16700	-8.8286864	0.01717854	-1.4927304	7.82083419	-4.7290324	0.00226243
Krtap19-5	16704	-8.7824723	0.00496434	1.50713662	7.63941681	-7.6160148	0.00013894
Krtap1-5	69664	-8.7505114	0.01378664	-0.997786	8.14260526	-5.1397528	0.00143015
Krtap14	23927	-8.6939702	0.00316309	3.08367099	7.48425057	-9.6534551	3.10E-05
Krtap19-3	77918	-8.6697807	0.00563244	1.11208836	7.54408447	-7.1722469	0.00020125
Krtap8-1	16703	-8.6489601	0.02374361	-2.1253491	8.8819581	-4.2320564	0.0040708
Krtap9-3	75586	-8.5110047	0.00653534	0.74067679	6.82155123	-6.7755114	0.00028474
Krtap19-9b	170939	-8.4136381	0.00968429	-0.1886954	7.97914031	-5.8598641	0.00067568
Krtap4-16	435285	-8.4012429	0.00775565	0.31532947	8.31664711	-6.3435469	0.00042312
Krt86	16679	-8.3569935	0.00604156	0.90698192	7.82105647	-6.9508062	0.0002438
Krtap19-1	170657	-8.166549	6.79E-05	9.07850447	6.41772797	-26.542816	3.65E-08
Krtap6-3	100040249	-8.1323025	0.01917858	-1.717057	7.64739149	-4.5494409	0.00278576
Krtap13-1	268905	-7.9384286	0.0032761	2.91933469	7.76212006	-9.4183188	0.0000363
Krtap4-2	68673	-7.895653	0.00813145	0.18926149	7.49781985	-6.2198137	0.00047573
Krt25	70810	-7.6427047	0.01487684	-1.1800638	9.03489	-4.9860674	0.00169325
Krt15	16665	-7.5270649	0.00543544	1.21208836	7.64405447	-7.2242439	0.00030125
Krt31	16660	-7.3526068	0.00387892	2.37256186	7.65778724	-8.6766108	6.13E-05
Gm11567	670533	-7.3074699	0.0003803	6.79637061	7.3869069	-17.251122	6.72E-07
Krt27	16675	-7.281297	0.01454891	-1.1247913	8.93426855	-5.0323619	0.00160872
Krtap19-4	170654	-7.2238368	0.00060736	6.14503259	6.86074188	-15.486565	1.38E-06
Tchh	99681	-7.2212843	0.01917858	-1.7188151	7.92831879	-4.5480486	0.00279031
Krtap6-2	16701	-7.1602903	0.0210754	-1.9007795	7.41049708	-4.4051682	0.00330387
Krt71	56735	-7.0484731	0.008937	0.01997858	9.00077848	-6.0565985	0.00055673
Krt72	105866	-6.9591439	6.79E-05	9.91351032	7.76402535	-32.353411	9.50E-09
S100a3	20197	-6.9272543	0.00105445	5.39407735	7.36052524	-13.735941	3.08E-06
Krt85	53622	-6.6914978	0.0032761	2.90242896	8.39258228	-9.3944633	3.69E-05
Krtap3-3	66380	-6.6382159	0.00602672	0.92223434	6.55417048	-6.9670712	0.00024035
Krt33b	16671	-6.6286974	0.00248581	3.6235658	8.37906556	-10.46984	1.83E-05
Krtap21-1	170656	-6.6038864	0.02604837	-2.2915079	7.97494584	-4.1061328	0.00475172
Krt35	53617	-6.5822013	0.00053237	6.4584659	7.25348984	-16.303392	9.82E-07
Psors1c2	57390	-6.4834988	0.00070678	5.93466802	7.46922801	-14.968566	1.74E-06
Padi3	18601	-6.2118286	0.0003803	6.82900508	7.01052367	-17.346674	6.48E-07
Crisp1	11571	-6.180764	6.79E-05	9.71578694	5.22126093	-30.769464	1.34E-08
Gprc5d	93746	-6.0587842	0.0003803	6.81171227	7.50153235	-17.295949	6.60E-07
Crym	12971	-5.9986291	6.79E-05	9.4672708	7.14505227	-28.980268	2.01E-08
Sprr2h	20762	-5.84495	0.00022387	7.76108261	6.89168403	-20.435132	2.15E-07
Pinlyp	641361	-5.7089919	0.0283954	-2.4666502	8.15252207	-3.9752717	0.00559442
Gjb2	14619	-5.7053977	0.00442462	1.91964193	8.55678253	-8.1054918	9.42E-05
Ly6g6d	114654	-5.3992728	0.00129865	4.95224474	6.68105212	-12.821069	4.86E-06
Krt75	109052	-5.3761687	0.00316309	3.07576106	8.02108335	-9.6420008	3.12E-05
Fbp1	14121	-5.2872102	0.0003803	6.84902964	7.10057383	-17.405674	6.33E-07
Krtap28-13	71386	-5.237678	0.00249815	3.56659008	6.56189178	-10.380338	1.94E-05
Krt81	64818	-5.1872723	0.00345122	2.68065157	8.80600429	-9.0870969	4.57E-05
Sprr1b	20754	-5.1805402	0.00030922	7.23716925	5.73339882	-18.608216	4.04E-07
5430421N21	100126226	-5.1498534	0.00129865	4.94636452	8.39000264	-12.809399	4.89E-06
Krtap12-1	16694	-4.9402914	0.00016372	8.15445148	6.13592859	-21.995004	1.31E-07
Krtap3-1	69473	-4.6536851	0.00476803	1.68092716	8.14275807	-7.8188478	0.00011797
Gjb6	14623	-4.6484227	6.79E-05	9.14834594	6.31974171	-26.952578	3.29E-08
Krtap5-2	71623	-4.5253687	0.00129865	4.99919555	5.61745673	-12.914706	4.63E-06
Sptssb	66183	-4.43117	0.00060736	6.1348964	5.22608732	-15.461071	1.40E-06
Krtap5-4	50775	-4.3509852	0.0003803	6.89137876	6.10848111	-17.531388	6.03E-07
Cryba4	12959	-4.1570885	6.79E-05	9.15505162	6.01232053	-26.992521	3.26E-08
Krt82	114566	-4.1226182	6.79E-05	9.38345648	5.2812735	-28.420612	2.30E-08
Pmel	20431	-4.1214813	0.00022387	7.7284363	7.8307095	-20.313504	2.24E-07
Krt32	16670	-4.064303	0.00175114	4.48771818	7.03538396	-11.936582	7.79E-06
Krtap5-1	50774	-4.0233897	0.00180648	4.22432748	6.94609227	-11.466524	1.01E-05
Padi1	18599	-4.0041111	0.00303117	3.2118217	6.2112009	-9.8409986	2.74E-05
Tyrp1	22178	-3.9746449	0.00016372	8.10979393	7.54812763	-21.808571	1.38E-07
Tyr	22173	-3.950363	0.00012467	8.46945137	5.58278742	-23.38713	8.62E-08
Msx2	17702	-3.7229744	0.00230811	3.757189	6.2940312	-10.683023	1.61E-05
Capn12	60594	-3.4927592	6.97E-05	8.96992145	6.77152474	-25.927486	4.28E-08
Trpm1	17364	-3.4857377	0.00093347	5.58873006	5.38635127	-14.16443	2.51E-06
Hoxc13	15422	-3.4569443	0.00345128	2.65185897	6.03843264	-9.0479376	4.69E-05
Serpinb11	66957	-3.4233768	0.00678815	0.64647263	6.60724276	-6.6778497	0.00031088
Krt84	16680	-3.3333739	0.01924178	-1.7249652	6.3268073	-4.5431798	0.00280628
Sprr1a	20753	-3.2402857	0.00905816	-0.0146628	11.1367973	-6.0236033	0.00057492
Ptgds	19215	-3.1809031	0.00027882	7.48733498	6.47768665	-19.448533	3.00E-07
Dct	13190	-3.1420919	0.00076913	5.79889325	9.02441882	-14.646159	2.01E-06
Atp12a	192113	-3.0696148	0.02245014	-2.0161871	7.65682389	-4.3157735	0.00367786
Slc40a1	53945	-3.0093546	0.00029026	7.39586443	8.56122995	-19.134865	3.35E-07
Dusp14	56405	-2.9973813	0.00115149	5.26014918	8.4277102	-13.450447	3.54E-06
Dlx3	13393	-2.9972359	0.00499216	1.48290572	6.76464469	-7.5881139	0.00014214
Ggt1	14598	-2.9471938	0.00029026	7.34198175	5.56385584	-18.953607	3.57E-07
Bnc1	12173	-2.9369059	0.01417349	-1.0622794	7.01387758	-5.085041	0.0015182
Pla2g2e	26970	-2.9361919	0.00124377	5.12564996	6.85519011	-13.171022	4.07E-06
Car6	12353	-2.8923286	0.00949985	-0.1354287	9.03049252	-5.909626	0.00064311
Bambi	68010	-2.8571454	0.00124377	5.09330283	7.52853275	-13.104876	4.21E-06
Fhod3	225288	-2.7697668	0.00053722	6.41339237	7.65652393	-16.182414	1.03E-06
Alox8	11688	-2.7465289	0.0027572	3.40624753	5.28668926	-10.132812	2.27E-05
Oca2	18431	-2.7244355	0.0005748	6.31641376	5.51533515	-15.926208	1.15E-06
Capn8	170725	-2.700174	0.00114369	5.29109359	6.41647152	-13.515757	3.43E-06
Dsc2	13506	-2.6508519	0.00499216	1.47585445	7.00755987	-7.5800119	0.00014308
Cd34	12490	-2.5507414	0.00432557	-1.5670492	5.45660038	-13.450447	0.00000354
Foxn1	15218	-2.4996394	0.00371081	2.51558412	5.89367439	-8.8648519	0.0000535
Gabrp	216643	-2.4493572	0.00337858	2.77843329	6.50495463	-9.2213503	4.16E-05
Elf5	13711	-2.4246661	0.00171495	4.57695696	5.65096848	-12.100821	7.12E-06
Slc39a8	67547	-2.4074203	0.00171167	4.60167127	8.19004492	-12.146768	6.94E-06
Ahsp	170812	-2.4032746	0.00920666	-0.0578635	6.07174669	-5.9826447	0.00059844
Padi4	18602	-2.3733842	0.0013684	4.85596651	5.90060039	-12.631577	5.36E-06

Upregulated genes							
Gene	EntrzId	logFC	adj.P.Val	B	AveExpr	t	P.Value
Bst2	69550	1.50021587	0.01471391	-1.1459269	8.23732827	5.01462805	0.00164053
Pvalb	19293	1.50051682	0.00358752	2.57082611	12.4884844	8.93862493	5.07E-05
Lcp2	16822	1.50074925	0.02990395	-2.5785375	7.4661842	3.89262589	0.00621033
Capn3	12335	1.50116162	0.00371081	2.51293382	6.99358166	8.86132769	5.36E-05
Vapb	56491	1.50199133	0.00325172	3.02708799	8.69406292	9.57182447	3.27E-05
Itgb2	16414	1.50211456	0.01923983	-1.7242883	9.42097271	4.54371551	0.00280452
Samsn1	67742	1.51035704	0.01229471	-0.7708148	5.31850326	5.33531453	0.00115896
Frzb	20378	1.51191117	0.00500357	1.45084504	5.96412614	7.55133767	0.00014649
Vcam1	22329	1.51234115	0.0032761	2.87304288	6.89256701	9.35314239	3.80E-05
Rtn2	20167	1.51243097	0.00397053	2.2651283	10.049234	8.53776736	6.79E-05
Myom1	17929	1.51387952	0.00474722	1.71423824	9.67025895	7.85828019	0.00011433
Tpm3	59069	1.51539822	0.03777653	-2.9945517	6.1576346	3.59128493	0.00916824
Paqr7	71904	1.51631328	0.00812437	0.19756038	8.7970431	6.2279008	0.00047207
Klk1	16612	1.51864363	0.04767464	-3.3856471	4.90444252	3.31542448	0.01325146
Adgrd1	243277	1.519818	0.00569462	1.09120473	7.64010294	7.14942841	0.00020522
Man1a	17155	1.51997293	0.00295871	3.28666933	8.88732243	9.95228133	2.55E-05
Cd68	12514	1.52507611	0.01866056	-1.663723	9.98522406	4.59178946	0.00265125
Lox	16948	1.52692623	0.02974693	-2.5642718	9.4388868	3.90312334	0.00612813
Plekho1	67220	1.53160859	0.00500357	1.4526287	7.48143733	7.55337948	0.00014624
Irf4	16364	1.5319628	0.00230811	3.80535475	8.30275705	10.7610169	1.53E-05
Rraga	68441	1.53265065	0.00379127	2.45378717	7.88693333	8.78303301	5.67E-05
Vdac1	22333	1.53385743	0.00230811	3.82759286	10.1420376	10.7972356	1.50E-05
Cox7a1	12865	1.53634519	0.00387892	2.37055805	9.4597817	8.67400132	6.14E-05
Cox8b	12869	1.53964081	0.00190385	4.11791593	10.9026652	11.2825895	1.13E-05
Lgmn	19141	1.54001122	0.02964511	-2.555769	9.73509772	3.9093856	0.00607967
2900097C17	347740	1.54255642	0.02638338	-2.3142962	6.45083665	4.08899967	0.00485367
Pfn1	18643	1.54312113	0.0049204	1.54490119	10.5495643	7.65968238	0.00013409
Ces1f	234564	1.54324348	0.04529077	-3.300378	5.66410046	3.37503164	0.01222624
Fbxo32	67731	1.54563284	0.00554625	1.16171182	7.33967565	7.22671957	0.00019212
Sema3c	20348	1.54606343	0.00394284	2.32039045	8.1676288	8.60891634	6.44E-05
Rhoc	11853	1.54718413	0.00394284	2.31335241	7.51828028	8.59982336	6.48E-05
Tsc22d3	14605	1.54875332	0.0475661	-3.3813846	10.3024029	3.31839762	0.01319819
Cd209g	70192	1.55143906	0.01225033	-0.7498369	9.99573055	5.35363231	0.00113665
Tspan32	27027	1.55608316	0.02773934	-2.4089283	6.38431982	4.01819452	0.0053012
Tfec	21426	1.56193404	0.02447703	-2.1817563	6.04843335	4.18910812	0.00429016
Daglb	231871	1.56310838	0.02394583	-2.1415584	7.60828071	4.21969331	0.00413265
Myoz1	59011	1.56600677	0.00439501	1.94436902	11.3652579	8.13573259	9.20E-05
Ncf1	17969	1.56732617	0.03463548	-2.8442734	7.96315749	3.69911427	0.00796291
Trim54	58522	1.57406224	0.00181705	4.20480881	8.56065639	11.432535	1.03E-05
Dok2	13449	1.57748131	0.00769172	0.33335651	8.19548258	6.3613959	0.00041609
Igfbp3	16009	1.58068023	0.00624698	0.82371722	10.0195638	6.86257412	0.00026351
Fcna	14133	1.5818781	0.00609398	0.87185594	10.4132885	6.91346954	0.00025193
Serpinh1	12406	1.58405497	0.0029797	3.27021848	10.8308344	9.92771028	2.59E-05
Cacna1s	12292	1.58474121	0.013166	-0.9164184	8.33206361	5.20931423	0.00132631
Mcoln2	68279	1.58642288	0.02434035	-2.172909	6.18988192	4.19583068	0.00425498
Sln	66402	1.58907002	0.00500357	1.44757247	8.66440398	7.5475927	0.00014694
Pdlim7	67399	1.59181274	0.0032761	2.85404148	9.35915084	9.32652171	3.87E-05
Vopp1	232023	1.59521866	0.00230811	3.72725045	6.37558419	10.6348544	1.66E-05
Zfpm2	22762	1.59563712	0.00731694	0.46422178	5.78350629	6.49216131	0.00036839
Csf2rb	12983	1.59973091	0.00703809	0.55693774	6.71878039	6.58609737	0.00033792
Cacnb1	12295	1.60678756	0.00384073	2.40674189	7.68939943	8.7212401	5.93E-05
Rassf2	215653	1.60843327	0.02984856	-2.5715905	6.53961713	3.89773639	0.00617016
Cpz	242939	1.61173277	0.00230811	3.73215727	7.20087345	10.642733	1.65E-05
Pip4k2a	18718	1.6168176	0.00888621	0.03908629	6.89040685	6.07485635	0.00054694
Des	13346	1.61836775	0.00481791	1.65677467	10.8279518	7.79036983	0.00012069
Mustn1	66175	1.62253973	0.00593467	0.97467903	7.10456993	7.02324306	0.00022885
Synpo2	118449	1.62299469	0.00325911	3.01152388	8.19803774	9.54949495	3.32E-05
Aif1	11629	1.62417411	0.01168477	-0.6319813	7.73801141	5.4573297	0.00101907
Kdelr3	105785	1.63221724	0.00468822	1.76147168	8.37963319	7.91450486	0.00010935
Itgam	16409	1.640723	0.02264811	-2.0379435	6.75057618	4.29902412	0.00375301
Gpc3	14734	1.64300901	0.02156377	-1.9483395	8.78981215	4.36821566	0.00345312
Gas6	14456	1.64691305	0.0210754	-1.9012389	11.7098559	4.40481054	0.00330528
Ptp4a3	19245	1.65470456	0.00230811	3.78315225	9.34574655	10.7249882	1.57E-05
Tcea3	21401	1.65554199	0.01069235	-0.4120959	7.57777022	5.65446867	0.0008312
C1qtnf3	81799	1.66957254	0.01324447	-0.9290777	8.01737406	5.19845218	0.00134196
Ptgr2	77219	1.67139582	0.00177098	4.31883117	6.05882725	11.632713	9.22E-06
P4ha1	18451	1.67532339	0.00524652	1.2971952	6.86149429	7.37727327	0.00016921
Fermt3	108101	1.68231459	0.02434035	-2.1727114	8.01661469	4.19598084	0.0042542
Fn1	14268	1.69967463	0.00490871	1.57529465	5.85539429	7.6949899	0.00013031
Tmem38a	74166	1.70408885	0.0032761	2.94691488	8.679416	9.45736918	3.54E-05
Tmem100	67888	1.70998813	0.00446702	1.85373562	8.12233202	8.02539944	0.00010023
Cfp	18636	1.7104681	0.01211602	-0.7028327	9.49521245	5.39482896	0.00108821
Lrmp	16970	1.71139155	0.00672843	0.68145149	6.99630012	6.71397657	0.0003009
Camk1	52163	1.71439351	0.00301321	3.2496007	8.06929127	9.89700494	2.64E-05
Mgl2	216864	1.71714914	0.00624698	0.8259877	7.87969819	6.86496757	0.00026295
Cacng1	12299	1.71917015	0.00776351	0.31081665	7.48209958	6.33908478	0.0004249
Il10ra	16154	1.73551399	0.01079786	-0.4315219	5.98418209	5.6368552	0.00084631
Cpeb1	12877	1.76345417	0.00175114	4.40194416	5.8761627	11.7811339	8.49E-06
Cap2	67252	1.77441538	0.00175114	4.44372467	6.9622581	11.8565606	8.14E-06
Plek	56193	1.78075356	0.01386719	-1.0107557	7.71496579	5.12872043	0.00144744
Epsti1	108670	1.78283371	0.02002149	-1.8027137	7.13001436	4.48187084	0.00301629
Bin1	30948	1.78405649	0.00335055	2.79465302	8.89274063	9.24381105	4.09E-05
Cmtm3	68119	1.78717839	0.00230811	3.75970631	9.20165838	10.6870843	1.60E-05
Stat5a	20850	1.7918926	0.0049204	1.55377301	5.64889895	7.66997352	0.00013298
Six1	20471	1.79259079	0.00415503	2.08635828	5.13051156	8.31143817	8.04E-05
Rp2	19889	1.80472191	0.00472773	1.74054342	5.72649912	7.88954747	0.00011153
Sgcg	24053	1.80564756	0.00394284	2.3309236	7.31654293	8.62254226	6.38E-05
Abcc9	20928	1.8121532	0.00316309	3.08577923	6.80311511	9.65651034	3.09E-05
Agtr2	11609	1.81517283	0.02171863	-1.9629334	6.75766068	4.35690852	0.00350027
Asb12	70392	1.81809218	0.00129865	4.96741972	6.85180816	12.8512439	4.79E-06
Crem	12916	1.82285903	0.01938128	-1.7403685	6.54850671	4.53099799	0.00284668
Ccl9	20308	1.87135183	0.0172968	-1.5095559	10.3066359	4.71542649	0.002298
Ptx3	19288	1.88215381	0.00643366	0.76644728	6.13345836	6.80243156	0.00027797
Ptpro	19277	1.89364312	0.01228498	-0.7636146	6.18820107	5.341597	0.00115125
Sepw1	20364	1.89973395	0.00066455	6.0214335	10.8813136	15.1794249	1.58E-06
Krt6b	16688	1.90207946	0.0182467	-0.1886954	5.43346854	-7.9026465	4.48E-06
Ppp1r14a	68458	1.90308948	0.00438582	1.95660759	7.43384851	8.15073903	9.09E-05

Chac1	69065	-2.3380354	0.00479326	1.66548229	8.49202138	-7.800626	0.0001197
Nfe2l3	18025	-2.3057784	0.00345128	2.65746476	7.82345707	-9.0555486	4.67E-05
Mycn	18109	-2.3049601	0.00175114	4.39863105	5.31171891	-11.775176	8.51E-06
Gpnmb	93695	-2.24886	0.00411359	2.13927171	8.53125807	-8.3778265	7.65E-05
Cenpf	108000	-2.24688	0.00096657	5.52882999	6.06395008	-14.030813	2.67E-06
Ctse	13034	-2.2374723	0.00254727	3.53657365	8.58558159	-10.333516	2.00E-05
Ctps	51797	-2.2312293	0.00177098	4.30070813	8.63400757	-11.600633	9.39E-06
Ace2	70008	-2.210187	0.00346397	2.64213868	7.65176218	-9.0347555	4.74E-05
Gsdma	57911	-2.2026122	0.00283455	3.3587617	7.98602277	-10.060713	2.37E-05
Krt36	16673	-2.1852516	0.01432213	-1.0823692	7.43596181	-5.0680737	0.00154672
Otub2	68149	-2.182648	0.0003803	6.8687486	7.41870939	-17.464051	6.19E-07
Dnase1l2	66705	-2.1676732	0.0032761	2.88178663	8.09323131	-9.365418	3.76E-05
Fxyd4	108017	-2.1647508	0.0049204	1.53851304	6.21004451	-7.6522799	0.0001349
Lhx2	16870	-2.1227786	0.01042441	-0.3572743	7.33835374	-5.7043857	0.00079002
Trim59	66949	-2.1134684	0.00071873	5.89031575	7.85283842	-14.862249	1.82E-06
Tgm3	21818	-2.0859673	0.00405403	2.22540871	6.41887389	-8.4869785	7.05E-05
Dsg2	13511	-2.0811643	0.01417349	-1.0610693	7.9066718	-5.0860642	0.0015165
Aspm	12316	-2.0750628	0.0032761	2.93048044	5.73872331	-9.4340801	3.59E-05
Tmem229b	268567	-2.0737996	0.00326937	2.99799489	7.77569434	-9.5301285	3.37E-05
Mki67	17345	-2.0700216	0.00451807	1.83357039	8.52416968	-8.0010407	0.00010216
Gatad2b	229542	-2.0551678	0.00811851	0.20511388	8.33423848	-6.2352686	0.00046877
Tjp2	21873	-2.0511731	0.00177098	4.30744838	8.602044	-11.612552	9.33E-06
Ass1	11898	-2.0444757	0.00489775	1.60723082	7.91289879	-7.7322475	0.00012645
Krt18	16668	-2.0126937	0.00524652	1.2939586	5.13717374	-7.373645	0.00016972
Dlx2	13392	-1.992994	0.00536175	1.20923834	5.13628483	-7.2792253	0.00018375
Lap3	66988	-1.979988	0.00059485	6.22136337	10.1404033	-15.680353	1.27E-06
Kif2c	73804	-1.9684906	0.00193691	4.0875724	5.36218929	-11.230747	1.16E-05
Ccnb2	12442	-1.9639552	0.00892195	0.02939859	6.94983235	-6.0655943	0.00055188
Corin	53419	-1.9516372	0.00490871	1.58058243	4.65635797	-7.7011476	0.00012966
Tgfa	21802	-1.9493854	0.00356989	2.58168154	7.45464204	-8.9531927	5.02E-05
Sfn	55948	-1.9429397	0.00409404	2.18423389	9.71888905	-8.434634	7.33E-05
Rnf149	67702	-1.9388032	0.00339133	2.7523996	9.40655832	-9.1854137	4.26E-05
Cdkn1a	12575	-1.9107739	0.01307703	-0.8950146	9.24437529	-5.2277128	0.00130027
Cdc20	107995	-1.9088002	0.00406269	2.21445291	7.92858534	-8.4730204	7.12E-05
Ncapg	54392	-1.9084293	0.00830407	0.14064738	5.24734059	-6.1726023	0.00049771
Zfp185	22673	-1.876419	0.00177098	4.34459055	8.37904302	-11.678484	8.99E-06
Lce3a	545548	-1.87471	0.0013684	4.87306683	4.53732711	-12.664988	5.27E-06
Cyp2s1	74134	-1.8637103	0.00175114	4.448173	6.80514989	-11.864624	8.10E-06
Bdh1	71911	-1.8599937	0.00124377	5.16079821	6.79591749	-13.243356	3.92E-06
Slc39a6	106957	-1.8468527	0.00102625	5.44553794	9.5291521	-13.84763	2.92E-06
Jmy	57748	-1.8455411	0.00311773	3.1192228	7.59619636	-9.7051105	2.99E-05
Gpr143	18241	-1.8433365	0.00175114	4.38716229	4.80859635	-11.75458	8.61E-06
Tm4sf1	17112	-1.8432075	0.00230811	3.72072788	10.3193633	-10.624391	1.67E-05
Epha4	13838	-1.8380985	0.0053522	1.21834388	6.7643843	-7.2893225	0.00018219
Kif11	16551	-1.8305248	0.00604156	0.9042138	5.20091699	-6.9478577	0.00024443
Dusp2	13537	-1.822837	0.00340738	2.71626953	6.61689836	-9.1357724	4.41E-05
Sgol1	72415	-1.8191265	0.00344293	2.6965307	6.42520051	-9.1087653	4.50E-05
Psrc1	56742	-1.8136419	0.00129865	4.95524966	6.61251432	-12.827037	4.85E-06
Pthlh	19227	-1.8114765	0.00230811	3.78954575	5.76835784	-10.73535	1.56E-05
Nusap1	108907	-1.8047442	0.00124377	5.09786429	6.57264013	-13.114179	4.19E-06
Top2a	21973	-1.7991042	0.01898273	-1.6999399	8.0064598	-4.563009	0.00274186
Ccnb1	268697	-1.7863356	0.0090867	-0.0232478	6.70601172	-6.0154472	0.00057952
Cep55	74107	-1.772537	0.00439501	1.94795194	5.6208938	-8.1401232	9.17E-05
Bub1	12235	-1.767583	0.01182839	-0.6518024	5.52811884	-5.4397955	0.00103796
Spint1	20732	-1.7659078	0.00937068	-0.0994005	7.61205343	-5.9434597	0.00062196
Guca1b	107477	-1.7521538	0.00375721	2.47785152	5.76815773	-8.8148059	5.54E-05
2810417H13	68026	-1.7517512	0.00669262	0.68901957	8.73326082	-6.7218141	0.00029879
Pbk	52033	-1.7400266	0.00564303	1.10549621	7.68016306	-7.1650372	0.0002025
Stk26	70415	-1.7275888	0.00474722	1.72198838	7.11600453	-7.8674805	0.0001135
Uox	22262	-1.7148746	0.00395025	2.30160009	4.80407542	-8.5846603	6.56E-05
St14	19143	-1.7144306	0.00248703	3.59776294	8.39266901	-10.429205	1.88E-05
Slc5a8	216225	-1.7115565	0.01314718	-0.90954	4.76702014	-5.2152223	0.00131789
Sox9	20682	-1.7114669	0.01719557	-1.4969417	7.68753266	-4.7256249	0.00227128
Ect2	13605	-1.7069222	0.0053522	1.22433697	5.9174407	-7.295975	0.00018117
Csdc2	105859	-1.6978172	0.01010235	-0.2750446	5.47310748	-5.7798478	0.00073202
Endou	19011	-1.6951622	0.00628551	0.81313103	8.47769441	-6.8514238	0.00026612
Phlda2	22113	-1.695006	0.00230811	3.79547821	5.18451297	-10.744974	1.55E-05
B3gnt5	108105	-1.6920566	0.0050698	1.35775444	5.03021034	-7.4454509	0.00015987
Tfap2a	21418	-1.6870382	0.00474722	1.69739343	8.19626862	-7.8383173	0.00011616
Vdr	22337	-1.6864446	0.00734782	0.44539917	8.34555139	-6.4732228	0.0003749
Hmmr	15366	-1.6762798	0.00490871	1.57351686	6.13259196	-7.6929206	0.00013053
Lpin3	64899	-1.674116	0.00224938	3.910809	6.98501526	-10.933956	1.38E-05
Sytl2	83671	-1.6705712	0.00194487	4.07058945	7.99290697	-11.201848	1.18E-05
Upp1	22271	-1.6630543	0.00180648	4.2310938	6.82118062	-11.478334	1.01E-05
Cacna2d3	12294	-1.6627505	0.00730787	0.46920442	5.09056008	-6.497182	0.00036669
Stra6	20897	-1.6617895	0.00457224	1.79691809	5.87980439	-7.9569408	0.00010575
Fgfr3	14184	-1.6444035	0.0018393	4.17126055	8.45965934	-11.37438	1.07E-05
Ccna2	12428	-1.6389159	0.0099163	-0.2456197	7.30024706	-5.8070249	0.00071232
Gja1	14609	-1.6366587	0.00180648	4.26591984	10.8162013	-11.539333	9.72E-06
Dsp	109620	-1.6308063	0.00333431	2.80949332	9.50762799	-9.2644098	4.04E-05
Prpf40a	56194	-1.6084697	0.01060809	-0.396895	7.94284772	-5.6682785	0.00081957
Atp2c2	69047	-1.6075152	0.02108483	-1.9027748	6.32257661	-4.4036148	0.00331
Shcbp1	20419	-1.6044982	0.0050698	1.36401796	6.90376441	-7.452534	0.00015893
Cldn4	12740	-1.6012158	0.00474722	1.71180211	7.70113212	-7.8553903	0.00011459
Kif20a	19348	-1.5930869	0.0217724	-1.9684687	5.67995768	-4.3526237	0.00351832
Slc16a6	104681	-1.5894001	0.01725163	-1.5042383	6.68086732	-4.7197241	0.0022867
Cdk1	12534	-1.5893416	0.01719557	-1.497219	7.33474137	-4.7254005	0.00227186
Ap1m2	11768	-1.5887892	0.00180648	4.22584082	7.82885552	-11.469164	1.01E-05
Fam26e	103511	-1.5832673	0.00797643	0.24791567	7.63145216	-6.2771458	0.00045049
Fads3	60527	-1.5717904	0.0086064	0.09233781	9.0956094	-6.1259593	0.00052055
Cdca5	67849	-1.5673195	0.00813145	0.18521392	6.39304389	-6.2158723	0.00047752
Ovol1	18426	-1.56707	0.00586909	1.03885458	7.00157912	-7.0925014	0.00021552
Tbx1	21380	-1.5648367	0.0135236	-0.963312	7.498852	-5.1691514	0.0013852
Pkp3	56460	-1.5600827	0.00410762	2.1507635	7.95251118	-8.3923111	7.57E-05
Med1	19014	-1.559927	0.00476803	1.68347182	7.93119602	-7.8218537	0.00011769
Pard6b	58220	-1.5584656	0.0053522	1.23020973	4.97509194	-7.3024989	0.00018018
Srms	20811	-1.5578932	0.0038163	2.41867397	6.44216104	-8.7368724	5.86E-05
Car2	12349	-1.5567473	0.00750708	0.39518721	6.90793818	-6.4229167	0.00039283
Elovl3	12686	-1.5557336	0.00583992	1.0551972	9.10206123	-7.110231	0.00021225
Acpp	56318	-1.5482891	0.00529319	1.26692737	8.50482077	-7.3434035	0.00017408
Fgf22	67112	-1.5432191	0.00415503	2.08724004	7.15459585	-8.3125404	8.04E-05
AI661453	224833	-1.538565	0.00609398	0.87219255	7.85673028	-6.9138265	0.00025185
Spc25	66442	-1.5187561	0.00536175	1.20975779	6.43358023	-7.279801	0.00018366
Sdc1	20969	-1.5148682	0.00526953	1.28255009	10.0172441	-7.3608683	0.00017155
Ncaph	215387	-1.5129984	0.00442929	1.90577882	7.28035835	-8.0885833	9.54E-05
Cited4	56222	-1.5117	0.00175114	4.49926188	7.51005002	-11.957682	7.70E-06
Cks2	66197	-1.5097838	0.00175114	4.41525925	8.39702889	-11.805112	8.37E-06

Fxyd2	11936	1.90474313	0.00483469	1.64335746	8.00239784	7.77459047	0.00012222
Serinc3	26943	1.91183997	0.00490871	1.58773359	5.41363834	7.7094822	0.00012879
Gpt2	108682	1.91894135	0.00230811	3.74895351	7.57122527	10.6697495	1.62E-05
P2rx4	18438	1.92305854	0.00910829	-0.0346313	8.25751677	6.00464518	0.00058568
Tnfrsf21	94185	1.92403888	0.00230811	3.81884652	7.55329808	10.7829748	1.51E-05
Coro1c	23790	1.93927747	0.02072228	-1.865208	5.9539826	4.43291085	0.0031965
Tnnt1	21955	1.94506386	0.00933683	-0.0857492	7.22581009	5.95631692	0.00061413
Pkia	18767	1.95269542	0.00175114	4.40287654	7.93168097	11.7828112	8.48E-06
Ap2a2	11772	1.95382662	0.00734782	0.44574156	7.91562126	6.4735669	0.00037478
Tdo2	56720	1.96278401	0.03643377	-2.9355136	5.11016673	3.63351573	0.00867406
Arrb2	216869	1.9775673	0.0053522	1.22016726	7.69333395	7.29134596	0.00018188
Car8	12319	1.99320038	0.04410285	-3.2572698	7.02915404	3.40527428	0.01173907
St3gal4	20443	1.99500919	0.00604156	0.90297432	7.28663064	6.94653784	0.00024471
Prrx1	18933	1.99937816	0.00410762	2.16997436	7.34403058	8.41657822	7.43E-05
Fabp3	14077	2.00643928	0.00397053	2.26266781	8.44784837	8.53461271	6.80E-05
Pecam1	18613	2.01395307	0.00059485	6.21738198	7.69769097	15.6701659	1.28E-06
Dab2	13132	2.06067995	0.00431675	2.01675422	7.98048441	8.22486552	8.59E-05
C5ar1	12273	2.09783209	0.01229471	-0.7684539	7.00368496	5.33737392	0.00115642
Cyp7b1	13123	2.13895724	0.00786383	0.28389658	7.17438219	6.3125183	0.00043567
Pdgfd	71785	2.20422866	0.01220521	-0.7418177	5.16459602	5.36064572	0.00112824
Ear3	53876	2.48404832	0.00703247	0.56269377	7.38024606	6.59196502	0.00033612
Pon1	18979	2.99662183	0.00230811	3.74508223	8.98395085	10.663516	1.63E-05
Hamp2	66438	3.81600321	0.00171167	4.59738339	9.50427234	12.1387821	6.97E-06

Supplementary Figure 2

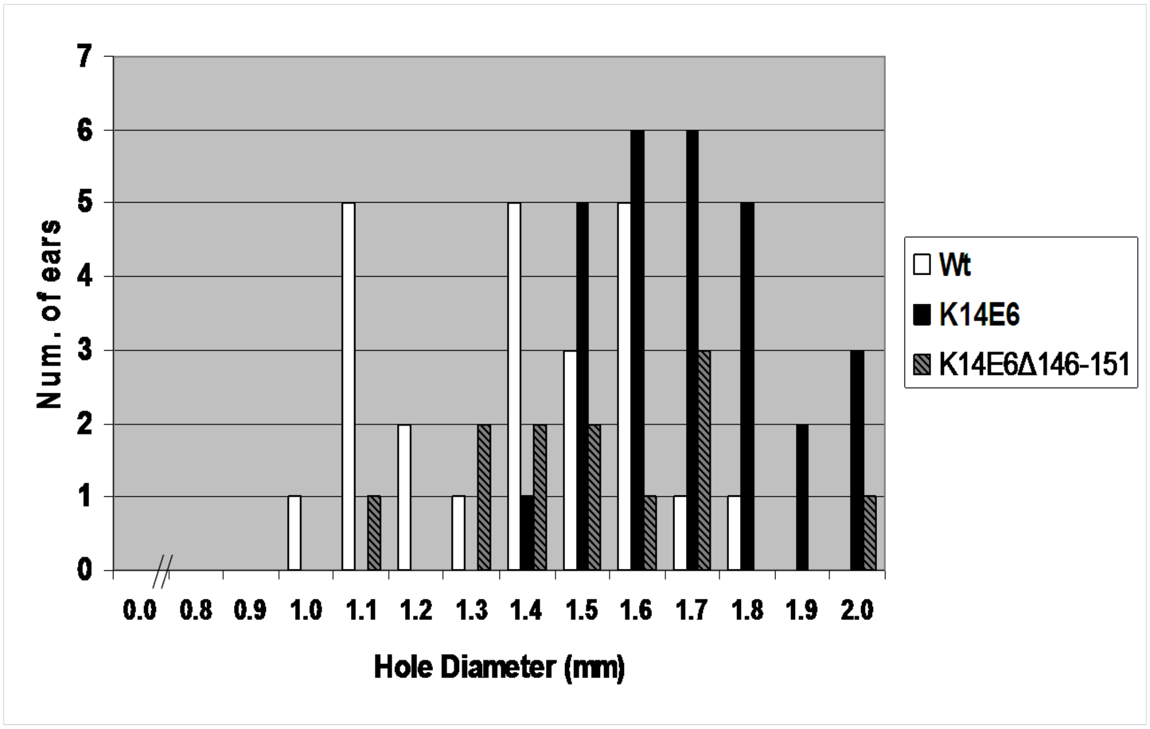

Supplement: Supplementary file 1 — Supplementary Figure 1 (Caption): A complete list of differentially expressed genes from K14E6 vs. K14E6d146-151 mice transcriptional profiles. Supplementary Figure 1 (Figure text): The list shows differentially up- or down-regulated genes resulted from global transcriptional microarray experiments. Supplementary Figure 2 (Caption): Histogram of wound closure of ears. Supplementary Figure 2 (Figure text): The histogram shows the distribution frequencies of wound closure every 0.1 mm in the ears of each mice strain. NTG: 24 samples, K14E6: 28 samples, and K14E6d146-151: 12 samples. [file 7868645.f1.pdf]
